# Editorial: Advances in mobile optical brain activity monitoring

**DOI:** 10.3389/fnrgo.2025.1568619

**Published:** 2025-03-07

**Authors:** Surjo R. Soekadar, Felix Scholkmann, Meryem Ayşe Yücel, Paola Pinti, J. Adam Noah, Alexander von Lühmann

**Affiliations:** ^1^Clinical Neurotechnology Lab, Department of Psychiatry and Neurosciences, Charité Campus Mitte (CCM), Charité – Universitätsmedizin Berlin, Berlin, Germany; ^2^Biomedical Optics Research Laboratory, Department of Neonatology, University Hospital Zurich, University of Zurich, Zurich, Switzerland; ^3^Institute of Complementary and Integrative Medicine, University of Bern, Bern, Switzerland; ^4^Neuroscience Center Zurich, University of Zurich and ETH Zurich, Zurich, Switzerland; ^5^Department of Biomedical Engineering, Neurophotonics Center, Boston University, Boston, MA, United States; ^6^Centre for Brain and Cognitive Development, Birkbeck, University of London, London, United Kingdom; ^7^Department of Medical Physics and Biomedical Engineering, University College London, London, United Kingdom; ^8^Department of Psychiatry, School of Medicine, Yale University, New Haven, CT, United States; ^9^BIFOLD - Berlin Institute for the Foundations of Learning and Data, Berlin, Germany; ^10^Technical University Berlin, Intelligent Biomedical Sensing Lab, Berlin, Germany

**Keywords:** mobile fNIRS, optical brain imaging, wearable neurotechnology, hyperscanning, neuroadaptive technology, real-world brain imaging, neuroethics

Advancements in brain imaging have significantly enhanced our understanding of brain function, but much of this progress stems from constrained, single-snapshot experiments conducted in controlled laboratory settings. Understanding brain activity in dynamic, complex, and multisensory real-world environments remains in its infancy. Emerging mobile brain imaging technologies beyond electrocencephalography (EEG) (Nann et al., 2019), such as functional near-infrared spectroscopy (fNIRS) (Boas et al., 2014) or diffuse optical tomography (DOT) (Chitnis et al., 2016), are beginning to bridge this gap, enabling continuous measurement of cerebrovascular activity linked to brain activity induced by, for example, human movement, perception, cognition, social communication, and interaction in naturalistic settings. For instance, portable fNIRS devices have proven effective for monitoring mental workload (Herff et al., 2013; Park, 2023) and can provide real-time feedback, e.g., in the context of brain-computer interface (BCI) applications (Soekadar et al., 2021). In education, fNIRS has been used to study attention (Harrivel et al., 2013), engagement (Verdiere et al., 2018), and learning outcomes (Lamb et al., 2022) in natural settings, while its role in infant development research has expanded understanding of perception and cognition in diverse populations (Gervain et al., 2023). Moreover, hyperscanning (Hakim et al., 2023; Scholkmann et al., 2013) enables simultaneous measurement of brain activity in multiple individuals, revealing mechanisms like inter-brain synchrony during social interactions. Integrating fNIRS with multimodal tools such as EEG (von Luhmann et al., 2017), eye-tracking (Isbilir et al., 2019), and systemic physiological monitoring (Scholkmann et al., 2022) enhances these insights, specifically into learning processes and interpersonal dynamics, paving the way toward new medical and non-medical applications.

The Research Topic “*Advances in Mobile Optical Brain Activity Monitoring*” underlines the transformative potential of portable fNIRS and related optical techniques for investigating brain function in real-world and dynamic settings. Featuring eight contributions from leading laboratories, this Research Topic highlights cutting-edge advancements in the field.

In her review, Klein underscores the critical importance of spatial specificity and signal quality in real-time fNIRS applications, which are vital for reliable data collection in neurofeedback and BCI contexts. Challenges such as anatomical variability, variations in cap placement, and contamination by extracerebral noise and motion artifacts are addressed, advocating for advanced preprocessing techniques and adaptive algorithms to improve reproducibility and reliability.

Biswas et al. present a novel low-cost approach to non-invasive blood flow monitoring with integrated Diffuse Speckle Contrast Spectroscopy (iDSCS). By leveraging a low-cost photodiode and a custom electronic circuit, iDSCS simplifies deep tissue blood flow measurements, offering a compact, power-efficient alternative to traditional Diffuse Correlation Spectroscopy (DCS). Their study demonstrates the feasibility of wearable probe-level hemodynamic blood flow monitoring.

Bonnaire et al. propose an innovative approach utilizing hyperscanning with fNIRS to study social bond formation in children. The study integrates multimodal data, including conversational behaviors, interpersonal rapport, collaborative tasks, and inter-brain synchrony. The findings aim to deepen understanding of social connectivity while informing the design of empathetic AI systems and personalized educational tools that adapt to group dynamics.

Moffat et al. advocate for mobile fNIRS in longitudinal and intergenerational hyperscanning studies to uncover the neural mechanisms underlying social dynamics across generations. By addressing ecological validity challenges with portable designs and real-world protocols, this perspective article specifically highlights interventions targeting intergenerational relationships, particularly in psychological and social contexts.

Roumengous et al. introduce the NIRSense Aerie, a wearable fNIRS system optimized for high-G environments encountered by military aircrew. The system monitors cerebrovascular oxygenation and perfusion during high-G-force exposure, offering real-time feedback to improve anti-G straining maneuver training and operational safety. Future advancements in miniaturization and comfort will extend its applications to other high-stress occupations.

Lingelbach et al. investigate workload-dependent hemispheric asymmetries in emotion-cognition interactions using an ecologically valid fNIRS setup. Their findings reveal lateralized prefrontal cortex activity influenced by emotional distractions and workload levels, with implications for optimizing focus and productivity in learning and work environments.

Srinivasan et al. demonstrate the importance of incorporating subject-specific information to enhance spatial accuracy in high-density diffuse optical tomography (HD-DOT) using fNIRS. By employing photogrammetry to identify optode placement, their study shows the extend of optode localization errors, particularly in motor cortex recordings that ranged at 27.4 mm in average. Their work underlines the importance of collecting subject-specific optode locations for all wearable NIRS experiments to achieve accurate results.

Finally, von Au et al. examine the neural activation patterns associated with different self-touch behaviors, identifying distinct hemodynamic responses in the prefrontal cortex during phasic and repetitive self-touch. This study shows that repetitive self-touch activates the orbitofrontal cortex and dorsolateral prefrontal cortex brain regions associated with self-regulation, more strongly than phasic self-touch, highlighting the stronger self-regulatory function of repetitive self-touch, the importance of using objective behavioral controls, and the need for future research on irregular self-touch in real-world environments.

Besides underlining the critical challenges that must be addressed to drive the field forward, this Research Topic illustrates the transformative potential of mobile optical brain imaging technologies. While it is essential to advance innovation in instrumentation, data analysis, and experimental design, future efforts must also prioritize interdisciplinary collaborations to fully realize the promise of this exciting new research domain. Importantly, online monitoring of functional brain activity enables dynamic, brain-state-dependent interaction using sensory or brain stimulation (Nasr et al., 2022) or human-computer interaction (neuroadaptive technology, passive BCI) (Zander and Kothe, 2011), offering opportunities to enhance learning, cognition, wellbeing or ergonomics. Establishing neurotech hubs and innovation ecosystems centered around robust academic-industry-clinical collaborations will be crucial for facilitating the rapid prototyping and exploration of such innovative solutions. In this context, it is imperative to ensure that neurotechnology serves as an enabler of human potential and development, rather than a tool for surveillance, coercion, or any application that undermines human freedom or rights (UNESCO, 2024). Embedding these advancements within a robust neuroethical framework is essential to safeguard their responsible use (Garden et al., 2019). With these efforts, mobile optical brain imaging offers a bright future, redefining how we work, learn, and interact with digital technologies.
